# Forecasting cardiovascular disease mortality using artificial neural networks in Sindh, Pakistan

**DOI:** 10.1186/s12889-024-21187-0

**Published:** 2025-01-04

**Authors:** Moiz Qureshi, Khushboo Ishaq, Muhammad Daniyal, Hasnain Iftikhar, Mohd Ziaur Rehman, S. A. Atif Salar

**Affiliations:** 1 Govt Degree College TangoJam, Hyderabad 70060, Sindh, Pakistan; 2 Ibn-e-Sina Medical University Mirpurkhas, Sindh, Pakistan; 3 Department of Statistics, Faculty of Computing, The Islamia University of Bahawalpur, Bahawalpur, Pakistan; 4 Department of Statistics, Quaid-i-Azam University, 45320, Islamabad, Pakistan; 5 Department of Finance, College of Business Administration, King Saud University, P.O. Box 71115, Riyadh, 11587 Saudi Arabia; 6 Al-Barkaat Institute of Management Studies, Aligarh 202122, Dr. A. P. J. Abdul Kalam Technical University, Lucknow 226010, India

**Keywords:** Cardiovascular disease, Analyzing and forecasting, Mortality, Time series models, Artificial neural network approach

## Abstract

Cardiovascular disease (CVD) is a leading cause of death and disability worldwide, and its incidence and prevalence are increasing in many countries. Modeling of CVD plays a crucial role in understanding the trend of CVD death cases, evaluating the effectiveness of interventions, and predicting future disease trends. This study aims to investigate the modeling and forecasting of CVD mortality, specifically in the Sindh province of Pakistan. The civil hospital in the Nawabshah area of Sindh province, Pakistan, provided the data set used in this study. It is a time series dataset with actual cardiovascular disease (CVD) mortality cases from 1999 to 2021 included. This study analyzes and forecasts the CVD deaths in the Sindh province of Pakistan using classical time series models, including Naïve, Holt-Winters, and Simple Exponential Smoothing (SES), which have been adopted and compared with a machine learning approach called the Artificial Neural Network Auto-Regressive (ANNAR) model. The performance of both the classical time series models and the ANNAR model has been evaluated using key performance indicators such as Root Mean Square Deviation Error, Mean Absolute Error (MAE), and Mean Absolute Percentage Error (MAPE). After comparing the results, it was found that the ANNAR model outperformed all the selected models, demonstrating its effectiveness in predicting CVD mortality and quantifying future disease burden in the Sindh province of Pakistan. The study concludes that the ANNAR model is the best-selected model among the competing models for predicting CVD mortality in the Sindh province. This model provides valuable insights into the impact of interventions aimed at reducing CVD and can assist in formulating health policies and allocating economic resources. By accurately forecasting CVD mortality, policymakers can make informed decisions to address this public health issue effectively.

## Introduction

Cardiovascular disease (CVD) is a broad term encompassing various heart and blood vessel conditions. It is a leading cause of death worldwide, accounting for an estimated 17.9 million deaths each year, according to the World Health Organization (WHO). The most common types of CVD include coronary artery disease, stroke, heart failure, and peripheral artery disease. These conditions can develop over time due to a combination of factors, including high blood pressure, high cholesterol, smoking, diabetes, obesity, physical inactivity, and a family history of heart disease (https://www.who.int). In Sindh, CVDs are a serious public health issue. Age, gender, obesity, hypertension, hyperglycemia, and hyperlipidemia are the main risk factors for cardiovascular disease. Nonetheless, insufficient is known about the frequency and risk factors related to Hyderabad's population in both urban and rural areas [1]. The burden of CVD is characterized by its immense prevalence, as it remains the leading cause of death globally. This encompasses the human toll in terms of lives lost and affected and the economic burden of managing and treating these conditions. In addition, early diagnosis and treatment are crucial for managing CVD and reducing the risk of serious complications. Besides unhealthy lifestyle choices, other risk factors for heart disease include smoking, alcohol, high cholesterol levels, obesity, high blood pressure, and diabetes [2]. According to the WHO, CVDs are a group of disorders of the heart and blood vessels. Collectively, CVD accounted for an estimated 17.9 million deaths worldwide in 2019. Of these, representing 32% of all global deaths. According to the most recent WHO data on the heart attack ratio in Pakistan, 240,720 people died from coronary heart disease in Pakistan in 2020, accounting for 16.49 percent of all fatalities. The intensity and ratio of deaths are increasing, which is dangerous for public health in Pakistan and Southeast Asia (https://www.who.int/data/gho/data/countries) [3, 4].

Modeling CVD cases is a multidisciplinary endeavor that leverages various approaches, from epidemiology and statistics to cutting-edge machine learning and artificial intelligence techniques. These models are instrumental in unraveling the intricate web of factors contributing to CVD, including genetics, lifestyle choices, environmental factors, and healthcare interventions. In this era of data-driven healthcare, CVD modeling approaches are at the forefront of efforts to predict, prevent, and manage this pervasive and life-altering disease. They offer valuable insights into disease trends, risk factors, and the effectiveness of interventions [5, 6]. Several models have been applied in the literature to model and predict the death rates due to CVD. Three distinct statistical models were employed to forecast heart disease, specifically utilizing a Support Vector Machine (SVM), Decision Tree (DT), and Logistic Regression model (LR) [7]. The investigation revealed that through the application of the 'C-Rule' and employing various combinations, it is possible to improve predictive accuracy [8]. A time series model was introduced, proposing a novel approach known as the combined reinforcement multitask progressive time series model for CHD prediction [9]. The findings indicated that deep reinforcement learning (DRL) pre-training and multitasking exhibited superior performance in CHD prediction. In another study, five machine learning models were harnessed to predict the daily admissions for cardiovascular disease (CVD) [10]. After subjecting them to key performance indicators for comparison, it was evident that the Random Forest (RF) model surpassed its peers in forecasting daily CVD admissions. To further enhance the forecasting of heart disease, a hybrid time series modeling approach was applied, combining the Support Vector Machine model (SVM) and Random Forest (RF) [11]. This hybrid model significantly boosted forecasting efficiency by up to 88.7%.

Moreover, the ARIMA model was deployed to predict the mortality rate of CVD patients, and its efficiency in this context was duly noted [12]. Researchers employed the Lee-Carter and Bayesian Age Period Cohort (BAPC) models for broader mortality trend projections, extending their forecasts to 2030 in England [13]. In the context of disease classification and forecasting, various machine learning models were applied, particularly for Romania's International Classification of Disease (ICT) [14], with the findings emphasizing the models' significance in this predictive task. The authors [15] made a comparative analysis based on linear and non-linear time series models to predict the stay at the ICU using a sample size of 6064. The data was thoughtfully partitioned into training and testing sets, ultimately revealing that the Gaussian Naive Bayes and Logistic Regression hybrid model (GB + LR) exhibited superior performance in predicting the overall survival of cardiac patients. A comparative evaluation to predict CVD based on machine learning and conventional logistics regression is examined [16]. Results showed that the machine learning models predict more accurately than the classical method. The authors [17] also used time series regression in epidemiology studies to investigate the short-term association. Hypertension is considered one of the significant risk factors in developing CVD, as studied by [18]. The study [19] aimed to explore the relation between the risk factors of myocardial infarction (MI), and to achieve this end, binary logistic regression is applied. The study found that gender, family history, and other related variables are statistically significant for MI. A survey of risk factors [20] found that multiple risk factors are statistically significant in developing CVD in Pakistan.

Moreover, the authors [21] conducted a cross-sectional study at NICVD on CVD knowledge. Logistic regression [22] is used and compared with machine learning models in predicting chronic disease. For further details on these studies, interested readers are encouraged to refe`r to the respective citations [23, 24].

The ARIMA and Seasonal-ARIMA are time series forecasting models used in statistics and econometrics to analyze and predict patterns in time-dependent data. ARIMA is significant because it provides a flexible framework for modeling and forecasting time series data, making it a valuable tool. At the same time, SARIMA allows for more accurate modeling and forecasting of time series data that exhibit both short-term fluctuations and longer-term seasonal patterns. Time series models, exemplified by ARIMA and SARIMA, find widespread utility in analyzing CVD incidence data and making short-term predictions. ARIMA models are the best-known model for time series forecasting and have been used by many researchers to predict infectious diseases with characteristic seasonal outbreaks [25, 26].

Almosova et al., [27] indicate the superiority of the machine learning model over than existing classical model in forecasting inflation variables. However, the studies in [28, 29] indicates the superiority of the ANNAR model over the classical approaches in modeling and forecasting the death of COVID-19 patients. Similarly, the article [30] has proposed ANNAR-based ensemble models for influenza incidents. The study found that the NNAR-based model results in the lowest accuracy error.

A study in Shandong, China, harnessed SARIMA modeling to aptly capture the seasonal and trend patterns in stroke incidence data, showcasing the model's ability to characterize such temporal dynamics effectively [31]. Given the shortcomings of ARIMA models, there is increasing interest in using ANN models for epidemiological time series forecasting [32] because these models account for nonlinearities in the data. Machine learning models, including artificial neural networks (ANNs) and support vector machines (SVMs), have emerged as valuable tools for CVD prediction. An A N-based approach, implemented in Shanghai, China, demonstrated superior predictive accuracy for stroke incidence compared to conventional time-series models [33].

This study aims to model and forecast annual mortality rates for CVD using various stochastic time series models by comparing the conventional linear and non-linear machine learning models. The paper is structured as follows: First, we provide a review of related literature review and previous work on CVD prediction. Furthermore, we provide details related to data description and methodology, followed by an overview of the results and their interpretation. Finally, we conclude with policy recommendations for future research direction.

## Materials and methods

The dataset in this study was collected from the Civil Hospital in the Nawabshah district of Sindh province, Pakistan. It includes actual cases of CVD related deaths that occurred between 1999 and 2021. A solid time series dataset that covers trends and patterns in CVD mortality over more than two decades is provided by this extensive collection, which contains yearly data. A thorough examination of the evolution of CVD-related mortality in response to numerous factors, including alterations in healthcare infrastructure, public health campaigns, lifestyle adjustments, and socioeconomic advancements in the area, is made possible by the dataset's wide temporal scope. Through the utilization of this abundant dataset, scholars can acquire a significant understanding of the epidemiology of cardiovascular disorders, pinpoint plausible risk factors, and formulate focused treatments aimed at alleviating the prevalence of CVD in Sindh province. This data set is free from any missing value and used with actual observations. Also, the ethical approval of this data set is received from the hospital administration.

### Statistical analysis

The data has been collected from Nawabshah, Sindh, Pakistan Civil Hospital, spanning the years 1999 to 2021, focusing on the number of people affected by CVD. To understand this data, which changes over time, a specific approach known as "time series analysis" is being employed. The analysis process involves several stages. Initially, the data will be subjected to a descriptive analysis to identify patterns and essential characteristics. This lays the foundation for subsequent phases. Following this, the data will be visualized by creating time series plots, enabling the observation of trends and changes in CVD deaths over the years. These visual representations help identify patterns such as seasonality and anomalies. Various time series models will be applied to make meaningful predictions and forecasts, each with its unique approach. These models include the straightforward "Naive" model, the more sophisticated "Simple Exponential Smoothing (SES)" method, "Holt's Linear Exponential Smoothing," and a highly advanced ANNAR Model." These models allow the data to be explored, patterns to be captured, and educated predictions about future trends in heart-related health issues to be made. The flow chart is given below for the data processing.
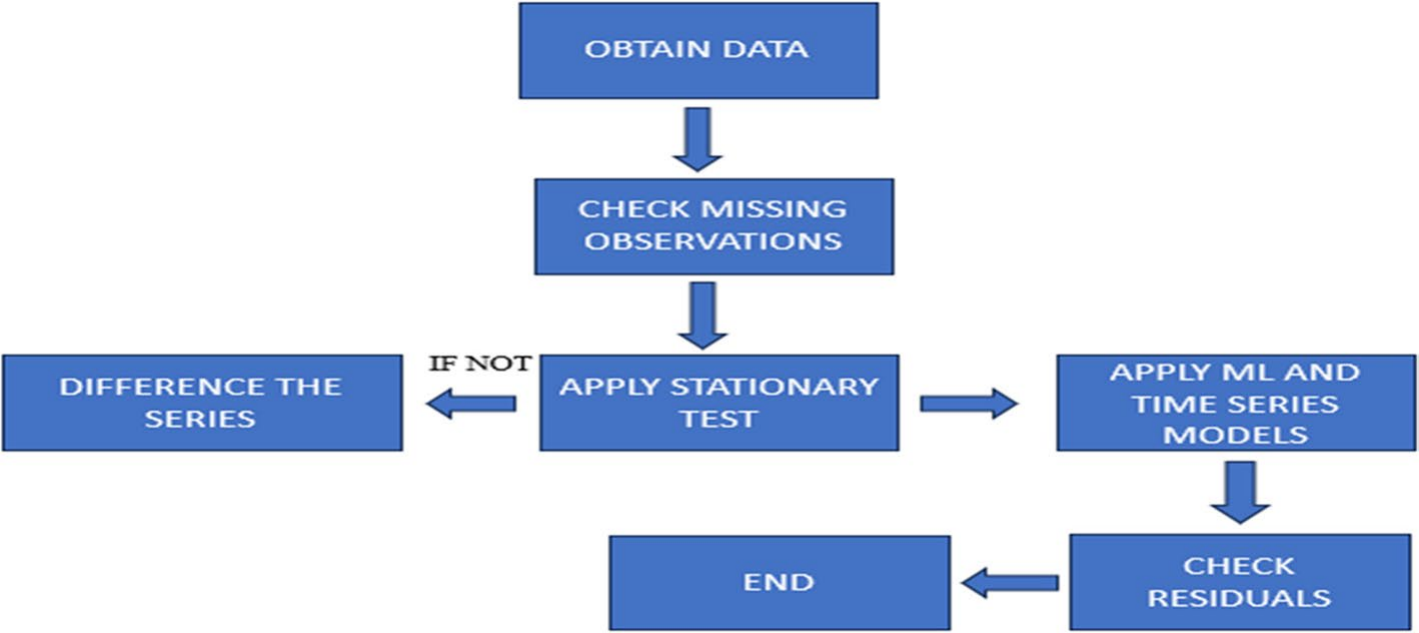


### Data description

**Fig. 1:**
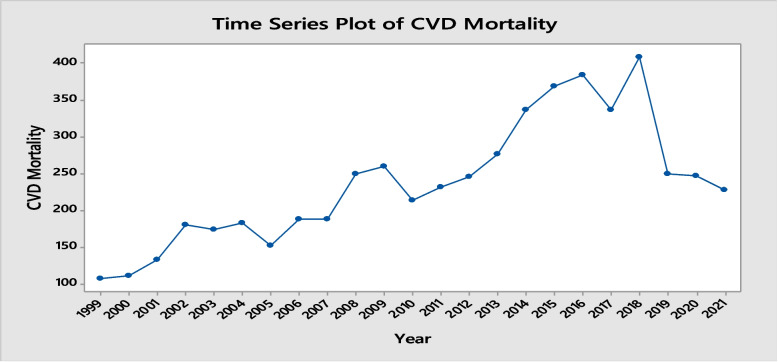
Time series of Yearly death cases of CVD

A time series plot was constructed using a graphical representation of data points collected over a period, then used to analyze and visualize changes in data over time. Figure 1 shows the visual display of the yearly time series of deaths from cardiovascular disease. Inspection of the time series plot in Fig. 1 suggests an increasing and decreasing trend.

The main focus of applying these models is to capture the data-generating process of the series. Both classical time series and machine learning models focused on short-term forecasting and then provided a comparison by indicator testing. The data was divided into 80% training (1999–2016) and 20% (2017–2021) testing for validation. The summary statistics are shown in Table 1 that the maximum number of death cases of CVD in Sindh was observed to be 107 in 1999, which continued to rise due to several factors until 2018 when the deaths rose to 408 cases. The average number of deaths was 236, with a median of 231. Figure 3 illustrates the residual diagnostic plots, which play a vital role in time series analysis as they help evaluate the adequacy of the time series model. Residuals in a time series model represent the discrepancies between observed values and the predicted values generated by the model. These plots are employed to visually assess whether the residuals adhere to certain assumptions: normal distribution, homoscedasticity (constant variance), and independence (lack of autocorrelation). The Histogram of residuals is used to verify the normal distribution of residuals. Additionally, the R function checkresiduals() is utilized to perform these diagnostic checks, generating a time plot, an ACF plot, a histogram of the residuals, and a normal curve.

**Table 1:** Summary statistics of CVD death cases

Minimum	107.0
Maximum	408.0
1st Quartile	181.0
3rd Quartile	268.0
Median	231.0
Mean	236.6
Skewness	0.42
Kurtosis	−0.79

In the fields of time series analysis and artificial intelligence, greater caution must be taken when working with small sample sets to ensure robustness and prevent overfitting. Below the methods are mentioned that highlight their use and robustness.

### Naïve method

In time series analysis, a naive method is the simplest forecasting approach where the next value in the time series is predicted to be equal to the current value. It can be expressed using the following equation:

1 $$\widehat y(t+1)=y(t)$$

where $\widehat y(t+1)$ represents the predicted value for the next time period, and $y(t)$ represents the actual value for the current time period. The naive method assumes that the time series is stationary and there are no trends or seasonal patterns in the data. It is a useful benchmark for evaluating the performance of more advanced forecasting models. The Naïve method makes minimal assumptions about the data and Less likely to overfit due to its simplicity [34, 35].

### Holt-winter exponential smoothing method

Holt-Winters forecasting, also known as triple exponential smoothing, is a popular time series forecasting method that uses exponential smoothing to capture trends and seasonality in the data. This method can handle small sample sizes effectively, but careful tuning of parameters is required to avoid overfitting [36]. The method involves using three smoothing equations, one for the level, one for the trend, and one for the seasonality, to produce a forecast.

The equations are:

2 $$\mathbf{L}\mathbf{e}\mathbf{v}\mathbf{e}\mathbf{l}\mathbf{e}\mathbf{q}\mathbf{u}\mathbf{a}\mathbf{t}\mathbf{i}\mathbf{o}\mathbf{n}:{T}_{t} = \alpha {Y}_{t}+ (1-\alpha )({L}_{t}-1 + {T}_{t}-1)$$

3 $$\mathbf{T}\mathbf{r}\mathbf{e}\mathbf{n}\mathbf{d}\mathbf{e}\mathbf{q}\mathbf{u}\mathbf{a}\mathbf{t}\mathbf{i}\mathbf{o}\mathbf{n}:{T}_{t}= \beta ({L}_{t} - {L}_{t}-1) + (1-\beta ){T}_{t}-1$$

4 $$\mathbf{S}\mathbf{e}\mathbf{a}\mathbf{s}\mathbf{o}\mathbf{n}\mathbf{a}\mathbf{l}\mathbf{e}\mathbf{q}\mathbf{u}\mathbf{a}\mathbf{t}\mathbf{i}\mathbf{o}\mathbf{n}:{S}_{t} = \gamma ({Y}_{t} - {L}_{t}) + (1-\gamma ){S}_{t}-m$$

5 $$\mathbf{F}\mathbf{o}\mathbf{r}\mathbf{e}\mathbf{c}\mathbf{a}\mathbf{s}\mathbf{t}\mathbf{e}\mathbf{q}\mathbf{u}\mathbf{a}\mathbf{t}\mathbf{i}\mathbf{o}\mathbf{n}:{F}_{t}+k = {L}_{t}+ k*{T}_{t} + {S}_{t}-m+1+k$$

where: ${Y}_{t}$ = the actual value at time $t$, ${L}_{t}$ = the level at time t, ${T}_{t}$ = the trend at time $t, {S}_{t}$ = the seasonal component at time $t$, $m$ = the number of seasons in a year $\alpha$, $\beta$, and $\gamma$ are smoothing parameters between 0 and 1, which control the amount of smoothing applied to each component. In forecast equation, ${F}_{t}$ represents the forecast for period t and k is the lag parameter or time shift i.e. how many periods ahead the forecast is made[37].

By estimating the smoothing parameters and applying these equations, a Holt-Winters forecast can be generated for future time periods.

### Artificial Neural Network Autoregressive (ANNAR) model

Neural network models are a type of machine learning model inspired by the structure and function of the human brain. They are designed to recognize patterns in data and make predictions or decisions based on that data. Neural networks consist of layers of interconnected nodes, called artificial neurons, inspired by the structure of neurons in the human brain. Each neuron receives input from other neurons, processes that input, and passes the result on to other neurons in the next layer.

Conventional time series models, such as exponential smoothing or ARIMA, assume that the data have linear connections. Nonetheless, a lot of time series from the real world show intricate, non-linear patterns. ANNAR models are good at capturing these complex interactions because of their non-linear activation functions [38]. ANNAR models are very adaptable and have a broad variety of function approximations. With sufficient data and processing power, they can model any underlying process thanks to their universal approximation capacity. ANNAR models can learn and identify patterns in the data without explicit specification of the model form [39].

**Fig. 2:**
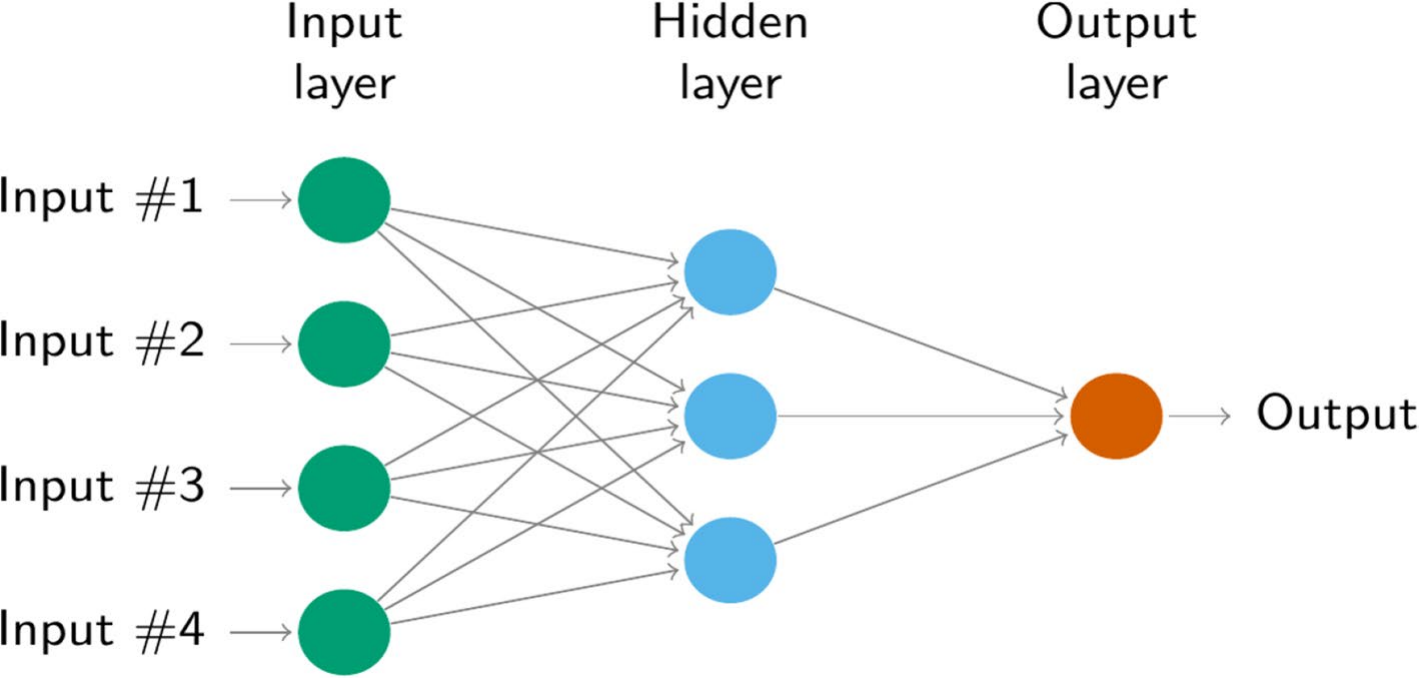
ANNAR model with four inputs one hidden layer with three hidden neurons

Neural network models have been used in a variety of applications, such as image and speech recognition, natural language processing, and game playing, amongst other uses. ANNAR models have been employed to predict disease outbreaks, patient admissions, and other health metrics. Their effectiveness in handling complex, multi-factorial data makes them suitable for these applications [40, 41]. They are a powerful tool for solving complex problems and have achieved state-of-the-art performance on many tasks [42, 43]. This network methodology permits to model of any linear and non-linear phenomena. Neural network autoregressive models are a type of model that is based on a simple neuronal structure that is organized in layers. These neural networks are classified further into two categories. The first category consists of the simplest neural network and the second category is a complex neural network. No hidden layer is involved in a simple neural network, while in a complex network, more than one hidden layer is used. In these neural networks, different methods are used to fit the data. The most commonly used procedure to fit the data is the feed-forward method. The graph of the feed-forward method is given in Fig. 2. The structure of a feed-forward network is composed of three parts, namely, the input layer that is used to process the observation, the hidden layer that is used to weigh these observations, and the output layer which is the gateway of the result. The hidden layer is responsible for processing being linked to a mathematical function according to suitable weight-age. The mathematical equation for the Neural Network autoregression can be written as.

6 $$f\left(w\right)= \sum\nolimits_{i=1}^{K}{W}_{i,j}{Y}_{i}$$

In Eq. 1 the variable ${Y}_{i}$ stands for the hidden layer algorithm which uses the sigmoid function. The sigmoid function is given in Eq. 7. Here ${W}_{i,j}$ represents the weight or coefficient associated with the ith element in the vector W of the jth element in the Y vector

7 $$g\left(y\right)= \frac{1}{1+{e}^{-y}}$$

The graph of the feed-forward method is given in Fig. 2.

### Simple exponential smoothing method

Simple exponential smoothing (SES) is a technique used for the forecasting of time series data. This method is usually applicable for forecasting of any time series. This method is assumed to fit best when the time series data has no seasonality or trend. This method assumes a weighting procedure for the successive time series observations, as it assigns weights in exponentially decreasing form over time. The mathematical formula of simple exponential smoothing can be written as [44].

8 $$f\left({s}_{t}\right)= \alpha {x}_{t}+\left(1-\alpha \right){s}_{t-1}$$

After simplification Eq. 8 results in

9 $$f\left({s}_{t}\right)={s}_{t-1}+\alpha ({x}_{t}-{s}_{t-1})$$

Here the

${\text{s}}_{\text{t}}=$ Smoothed statistic or the weighted average of current observation ${x}_{t}$

$${\text{s}}_{\text{t}-1}=\text{ One}-\text{time lagged smoothed statistic}$$

$${\alpha }=\text{ Smoothingparameterrangesfrom }0<{\alpha }<1$$

${\text{x}}_{\text{t}}$ = Current time period.

### Testing indicators

The most important task in time series analysis is the evaluation and selection of a suitable model. This is because the researcher assumes that the chosen model works more efficiently than others. Criteria exist for the application of a suitable model for forecasting, some of which are given below [45].

10 $$MSE= \frac{1}{n}{\sum }_{i=1}^{n}{e}_{t}^{2}$$

11 $$RMSE= \sqrt{\frac{1}{n}{\sum }_{i=1}^{n}{e}_{t}^{2}}$$

12 $$MAE= \frac{1}{n}{\sum }_{i=1}^{n}{e}_{t}$$

13 $$MAPE= \frac{1}{n}{\sum }_{i=1}^{n}\frac{|{e}_{t}|}{|{Y}_{t}|}*100$$

where ${e}_{t}$ stands for the error terms of yearly death cases and ${Y}_{t}$ stands for the observed time series at a point in time $t$. Based on these criteria, we select the model which results in the lowest number.

## Results

The study commenced by considering the number of modeling samples. It was determined that a sample size larger than 50 would be optimal for effectively capturing the statistical properties of the time series data. When the sample size is small, the parameters of the ARIMA model may become more inaccurate, leading to unreliable forecasts. In such instances, alternative methods such as simple exponential smoothing, naïve, and Holt-Winter models may be more appropriate [46, 47]. Simple exponential smoothing is particularly useful for forecasting time series data, especially when the sample size is small. This approach involves calculating weighted averages of past observations, with the weights gradually decreasing exponentially as the observations become older. To validate the model, the data was divided into an 80% training set and a 20% testing set.

It can be noted from Table 2, that in training 80% of the data the ANNAR model showed the lowest values of all KPIs. Since the observations are few and the rest 20% will be very few in numbers, we will be applying the rest of the techniques to the complete dataset. Neural networks can be applied when the sample size is small. In machine learning, neural networks can be applied to datasets of different sizes, ranging from a few data points to millions of data points. The size of the dataset does not determine the applicability of neural networks, but rather the complexity of the problem you are trying to solve and the architecture of the neural network [48, 49].

**Table 2:** Candidate models for the yearly CVD death cases Split data technique 80%

Candidate Models	MSE	RMSE	MAE	MAPE
Naïve	1790.14	42.31	33.23	14.11
SES	1690.85	41.12	31.39	13.32
Holt	1645.11	40.56	30.68	13.18
ANNAR	1572.12	39.65	29.31	12.70

Results from Table 3 show that the neural network autoregressive model (ANNAR) outperformed all the candidate models in testing on the complete dataset. The root means square error for the death case is 38.86 and the mean absolute error is 13.08 which indicates the dominancy of the ANNAR upon all the other selected models. The Naïve model showed the maximum values of KPIs as MSE = 2304, RMSE = 48, MAE = 33.86, and MAPE = 13.86 followed by the SES method showing MSE 2124.29, RMSE = 46.09, MAE = 33.85, and MAPE = 13.22. ANNAR showed the lowest values of KPIs among all candidate models applied and proved to be a better-performing methodology for modeling and forecasting the CVD death cases in Sindh. Further, we converted Table 2 into a visual form to enable a better understanding using residual analysis based on exploratory statistics. The residual plots of the death series and further showed the fitted versus the observed values of the CVD death cases.

**Table 3:** Candidate models for the yearly CVD death cases for 20% of data

Candidate Models	MSE	RMSE	MAE	MAPE
Naïve	2304.00	48.00	33.86	13.86
SES	2124.29	46.09	33.85	13.77
Holt	2082.09	45.63	32.213	13.22
ANNAR	1510.10	38.86	30.04	13.08

**Fig. 3:**
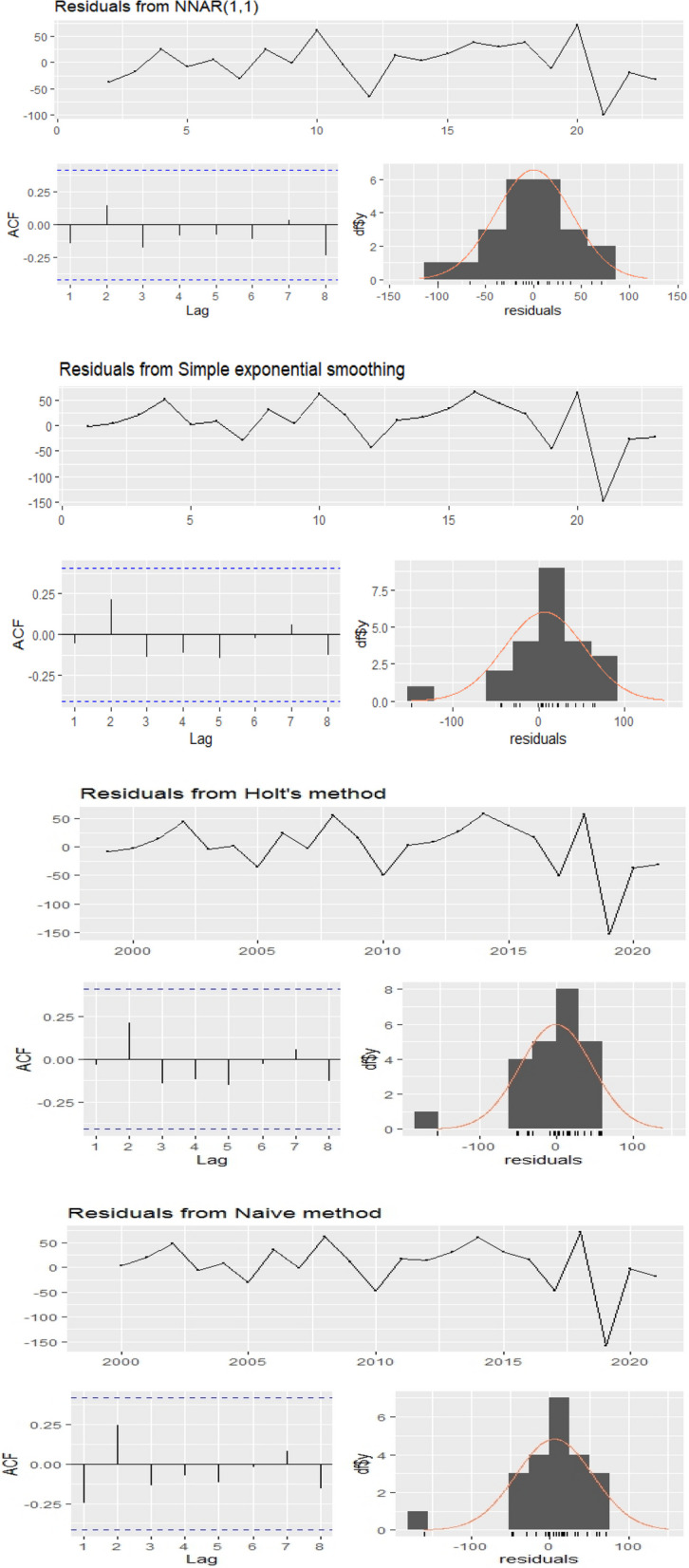
Residual Diagnostics of CVD death cases for ANNAR, SES, Holt, and Naïve

**Fig. 4:**
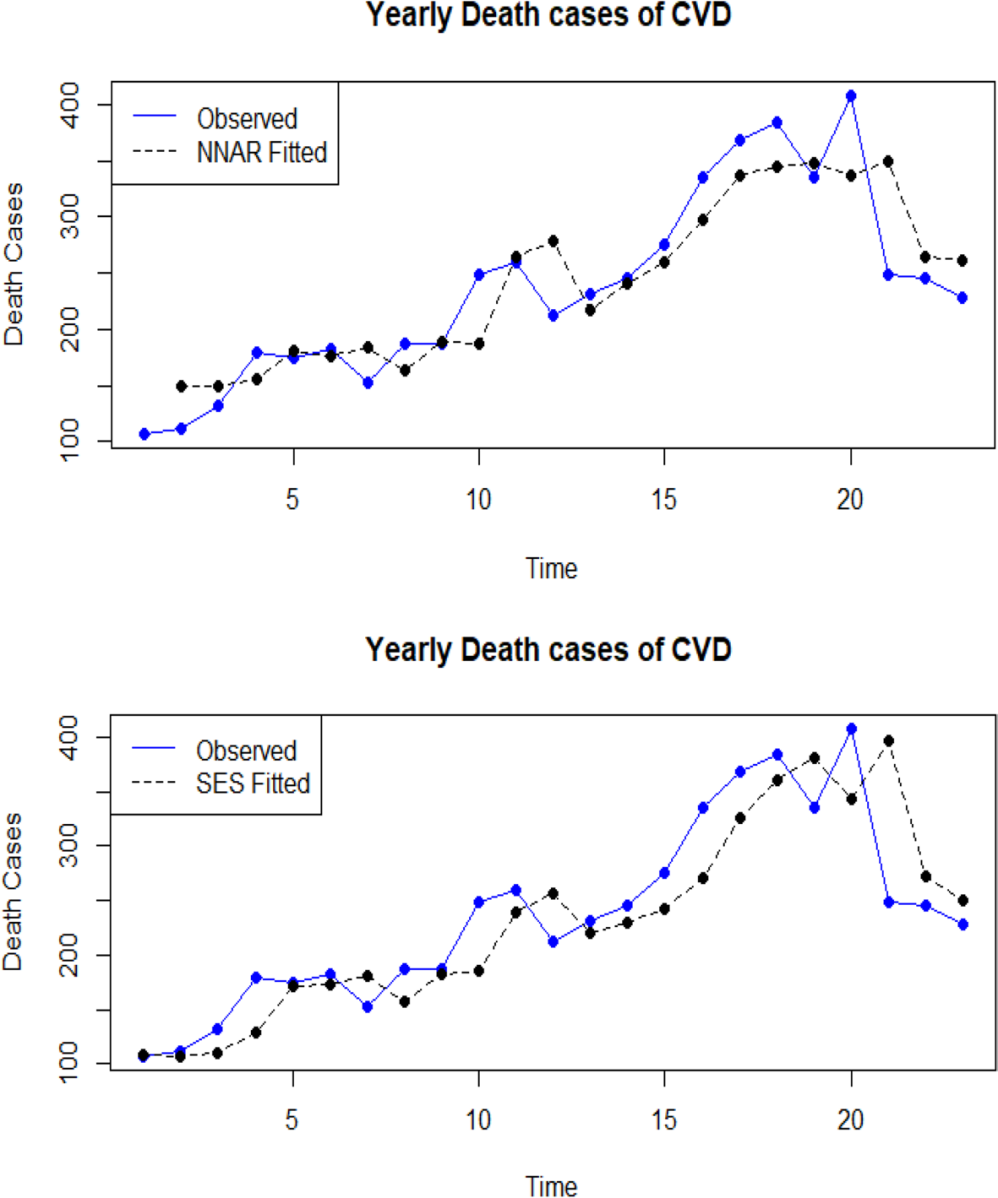
Observed versus fitted graph of CVD using ANNAR and SES

The Ljung-Box test is also conducted, wherein the null hypothesis assumes no autocorrelation among the residual terms, indicating a lack of model fit. The diagnostic examinations of the residuals concluded that the chosen ANNAR model exhibits a satisfactory fit without any autocorrelation among the residuals [47]. Figure 3 presents the diagnostic results, including the ACF plot and the plot of residuals overlaid with the normal curve, demonstrating the normality of the residuals. In a normally distributed scenario, the histogram should display a bell-shaped distribution, resembling the density plot. Notably, the histogram fitted using the ANNAR method aligns well with the residual data compared to other candidate models [50]. Furthermore, the lag values of the residuals generated by the ANNAR model fall within the acceptable probability limits. To further illustrate the closeness between observed and fitted observations, we present a plot depicting the observed versus fitted values of the series. Figure 4 shows the deviation from the observed is less through the neural network autoregressive approach which is an indication that this model is the best fit for the series. This is then used to make a next five-year forecast with the forecasted values given in Table 4 (95% confidence interval). Moreover the QQ-norm plots are given in Appendix.

**Table 4:** Long-term (5-year) forecasted values using neural network autoregressive

**Year**	Forecasted value	95% Lower C.I	95% Upper C.I
2022	236.32	158.13	314.29
2023	247.67	137.23	358.90
2024	263.06	122.28	384.12
2025	282.72	117.20	393.24
2026	304.09	120.54	392.08

## Discussion

In recent years, a model called the Artificial Neural Network Auto-Regressive (ANNAR) has become quite powerful [51, 52]. It's good at understanding complicated, non-straightforward connections in data. ANNAR has a track record of success in various situations, like predicting disease outbreaks and assessing the effectiveness of drugs, which has made it popular among researchers [53–55]. In this study, this work used traditional methods for analyzing time series data and more modern machine-learning techniques to the small sample size. Regarding the prediction of long-term cardiovascular disease cases, the ANNAR model outperformed the other techniques [56–58]. Further, this technique can be extended to trend analysis and other machine learning models based on a non-linear approach. The ANNAR model's improved performance is probably caused by its strong training and optimization strategies as well as its capacity to accurately capture and predict temporal dependencies and complicated, non-linear interactions seen in the data [59–61]. For a variety of time-series prediction tasks, its versatility, adaptability, and sophisticated feature processing make it a good fit [62–64].

In light of the results, it is recommended that preventive measures be taken to reduce the burden of CVDs in Sindh, including promoting healthy diets, increasing physical activity, and reducing tobacco use. Screening and early detection programs can also help diagnose and manage CVDs, reducing the risk of complications and improving outcomes. Individuals in Sindh need to take an active role in managing their heart health by making healthy lifestyle choices and seeking medical care when needed.

### Implication of the Study

The present study focused on modeling CVD death cases in the Sindh Province of Pakistan by conventional and non-linear time series models ANNAR. The outcomes demonstrated that the ANNAR model proposed in this research performed exceptionally well compared to the conventional methods. This study is unique in its nature as no such study has been performed for modeling the CVD death cases in the Sindh province of Pakistan. This study can help identify high-risk areas and populations that require greater attention and resources. This information can be used to prioritize resource allocation toward prevention and treatment strategies for those populations. Modelling CVD death cases can help estimate the economic impact of the disease in the region, and the cost-effectiveness of different interventions.

In the light of above suggestions, the following policy measures can be taken at the government level as well provincial level.

1. Encouraging healthy lifestyle choices: One of the best ways to prevent CVD is by adopting a healthy lifestyle, which includes regular exercise, healthy eating habits, quitting smoking, and reducing alcohol consumption. The government can launch public awareness campaigns to promote healthy living.
2. Providing access to preventive care: Early detection and treatment of CVD can significantly reduce mortality rates. Therefore, it is crucial to provide access to preventive care, such as regular check-ups, blood pressure and cholesterol screenings, and other diagnostic tests.
3. Improving the quality of healthcare services: Healthcare facilities in Pakistan need to be improved, and the quality of care should be enhanced. The government can invest in healthcare infrastructure, equip hospitals with modern technology, and train healthcare workers to provide better care.
4. Increasing taxes on unhealthy products: Taxes on unhealthy products, such as tobacco and sugary drinks, can reduce their consumption and promote healthier choices.
5. Providing access to affordable healthy foods: The government can encourage the production and consumption of healthy foods, such as fruits, vegetables, and whole grains, and make them more affordable for the general population.

These policies can significantly reduce the number of CVD deaths in Pakistan. However, implementing these policies requires a sustained effort and collaboration between the government, healthcare providers, and the public.

## Conclusion

Cardiovascular disease is one of the leading causes of death for humankind. This work aims to predict the yearly CVD patients in one Pakistani city Nawabshah, in the Sindh province. According to the findings, Sindh urgently needs focused public health programs to increase public knowledge of CVD risk factors such as tobacco use, physical inactivity, and unhealthy diets. For maximum effect, educational activities can be customized to target specific groups. In modeling diseases, there is no single method that is considered definitively better than all others. The choice of method depends on the specific requirements of the problem being addressed and the availability of data. Exponential models, such as simple exponential growth models, have been commonly used in the past to model the spread of diseases. These models are relatively straightforward to implement, but they have limitations when it comes to capturing complex disease dynamics. Neural network models, such as ANNAR, have gained popularity in recent years due to their ability to capture complex nonlinear relationships in data. ANNAR has been applied to a range of disease modeling problems, including predicting disease outbreaks and drug efficacy. This work applied different time series methodologies, one based on the classical time series method and the second based on the machine learning technique. The results found that the ANNAR outperformed for the long-term forecasting period.

Future research could focus on comparing a wider variety of modeling approaches outside of ANNAR and traditional time series methods. Identifying the best strategy for forecasting CVD death rates in Nawabshah, could involve utilizing additional machine learning models such as Random Forests, Support Vector Machines (SVM), and ensemble approaches.

## Figures and Tables

**Fig. 5:**
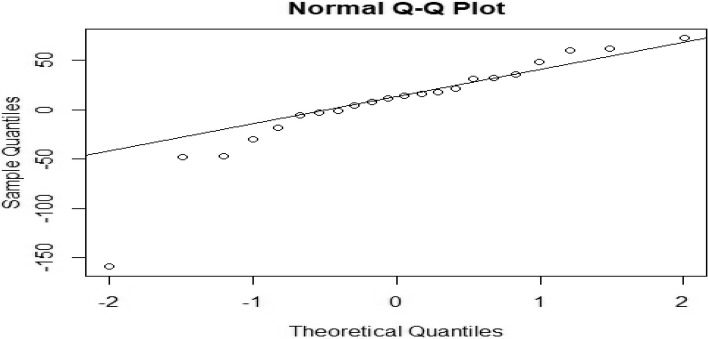
QQ norm Plot of Naïve method

**Fig. 6:**
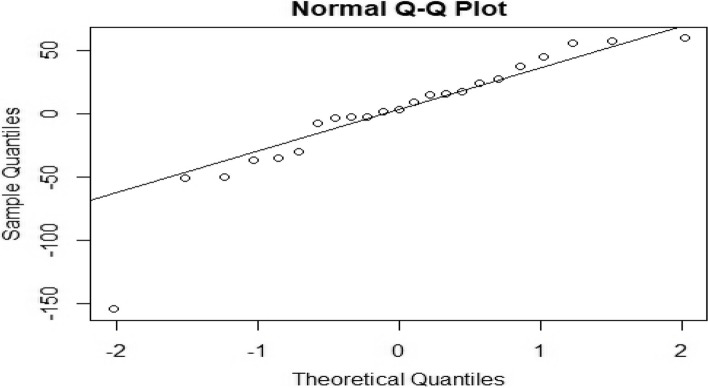
QQ norm Plot of Holts method

**Fig. 7:**
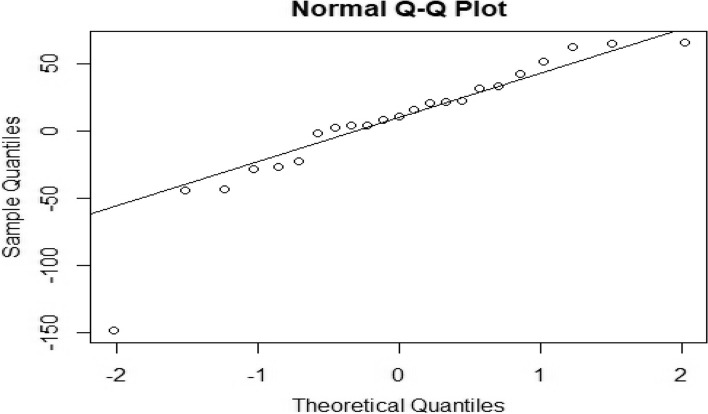
QQ norm Plot of SES method

**Fig. 8:**
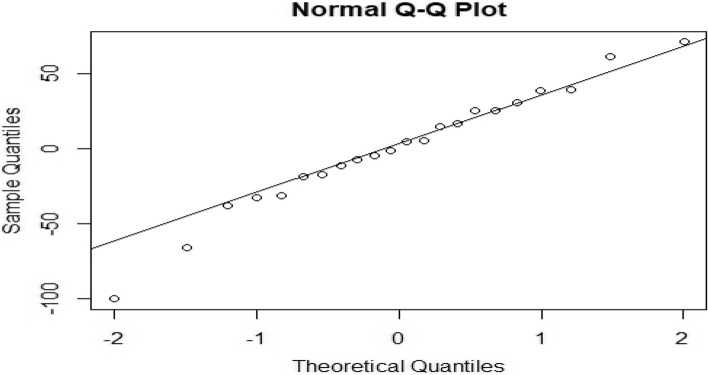
QQ norm Plot of ANNAR method

## Data Availability

The data that support the findings of this study are available from the corresponding author upon reasonable request.

## Appendix

5, 6, 7 and 8

## Abbreviations

CVD: Cardiovascular disease
ANNAR: Artificial neural network auto-regressive
SES: Exponential smoothing
ARIMA: Auto-regressive integrated moving average
SVM: Support Vector Machines
RMSE: Root Mean Square Deviation Error
MAE: Mean Absolute Error
MAPE: Mean Absolute Percentage Error
